# iPSC-derived human mesenchymal stem cells improve myocardial strain of infarcted myocardium

**DOI:** 10.1111/jcmm.12351

**Published:** 2014-06-28

**Authors:** Qingfeng Miao, Winston Shim, Nicole Tee, Sze Yun Lim, Ying Ying Chung, K P Myu Mia Ja, Ting Huay Ooi, Grace Tan, Geraldine Kong, Heming Wei, Chong Hee Lim, Yoong Kong Sin, Philip Wong

**Affiliations:** a National Heart Research Institute Singapore, National Heart Centre SingaporeSingapore; b Graduate Medical School, DUKE-NUSSingapore, Singapore; c Department of Cardiothoracic Surgery, National Heart Centre SingaporeSingapore; d Department of Cardiology, National Heart Centre SingaporeSingapore

**Keywords:** cell therapy, telocytes, myocardial strain, tissue deformation, myocardial compliance

## Abstract

We investigated global and regional effects of myocardial transplantation of human induced pluripotent stem cell (iPSC)-derived mesenchymal stem cells (iMSCs) in infarcted myocardium. Acute myocardial infarction (MI) was induced by ligation of left coronary artery of severe combined immunodeficient mice before 2 × 10^5^ iMSCs or cell-free saline were injected into peri-infarcted anterior free wall. Sham-operated animals received no injection. Global and regional myocardial function was assessed serially at 1-week and 8-week by segmental strain analysis by using two dimensional (2D) speckle tracking echocardiography. Early myocardial remodelling was observed at 1-week and persisted to 8-week with global contractility of ejection fraction and fractional area change in saline- (32.96 ± 14.23%; 21.50 ± 10.07%) and iMSC-injected (32.95 ± 10.31%; 21.00 ± 7.11%) groups significantly depressed as compared to sham control (51.17 ± 11.69%, *P* < 0.05; 34.86 ± 9.82%, *P* < 0.05). However, myocardial dilatation was observed in saline-injected animals (4.40 ± 0.62 mm, *P* < 0.05), but not iMSCs (4.29 ± 0.57 mm), when compared to sham control (3.74 ± 0.32 mm). Furthermore, strain analysis showed significant improved basal anterior wall strain (28.86 ± 8.16%, *P* < 0.05) in the iMSC group, but not saline-injected (15.81 ± 13.92%), when compared to sham control (22.18 ± 4.13%). This was corroborated by multi-segments deterioration of radial strain only in saline-injected (21.50 ± 5.31%, *P* < 0.05), but not iMSC (25.67 ± 12.53%), when compared to sham control (34.88 ± 5.77%). Improvements of the myocardial strain coincided with the presence of interconnecting telocytes in interstitial space of the infarcted anterior segment of the heart. Our results show that localized injection of iMSCs alleviates ventricular remodelling, sustains global and regional myocardial strain by paracrine-driven effect on neoangiogenesis and myocardial deformation/compliance *via* parenchymal and interstitial cell interactions in the infarcted myocardium.

## Introduction

Clinical evaluation of bone marrow-derived mesenchymal stem cells (BM-MSCs) in regenerative cardiac repair after myocardial infarction (MI) has been widely reported [1–3]. Despite early optimism in cellular replacement therapy, beneficial effects of BM-MSCs have largely been attributed to secreted paracrine factors that enhanced angiogenesis and modulated positive ventricular remodelling [4]. On the other hand, the seminal work of Popescu and colleagues has revealed telocyte as a distinct interstitial cell from fibroblasts [5] and other stromal cells [6]. Their close association with cardiac progenitors [7] and neoangiogenic endothelial cells [8] has increasingly been recognized to confer beneficial effects on human heart [9–11] that may have important implications in cardiac repair.

Compared to conventional BM-MSCs, alternative MSCs with superior proliferative potential and regenerative function have recently been derived from pluripotent stem cells such as human embryonic stem cells (ESC) [12] and induced pluripotent stem cells (iPSCs) by our group [13]. The ESC-derived MSCs have been reported to repair infarcted myocardium *via* paracrine effect comparable to adult-derived MSCs [14]. Moreover, angiogenic cytokines secreted from ESC-derived MSCs has also been reported as the main contributor in reducing ischaemia/reperfusion injury in a porcine model [15]. Bone marrow-derived MSCs are known improve global cardiac performance in dysfunctional myocardium majorly by ameliorating negative myocardial remodelling *via* restricting ventricular dilatation and enhancing neoangiogenesis [16]. Furthermore, BM-MSCs have been suggested to benefit cardiac performance by modulating tissue compliance and stress/strain response of infarcted myocardium [17]. Similarly, myocardial telocytes have been implicated in the repair of infarcted myocardium by recovering inter-cellular interactions *via* re-establishing connectivity and enhancing angiogenesis that benefited the restructuring infarct milieu [18]. Utilization of speckle tracking echocardiography (STE) in demonstrating myocardial strain may be helpful in delineating such re-established myocardial connectivity locally as STE affords regional quantification of structural deformational response of myocardial tissue in a multi-dimensional axis of radial, longitudinal and circumferential contractility that may be more informative in functional assessment after cardiac therapy [19].

It is unclear how localized transplantation of MSCs in a regional infarcted wall of left ventricle contribute to myocardial strain associated tissue deformation [20,21] and if telocytes have any role in myocardial compliance following MI. In this study, we investigated the global and regional myocardial strain of iPSC-derived MSCs (iMSCs) transplanted myocardium and associated myocardial telocytes by 2D strain analysis using STE.

## Materials and methods

### Generation and characterization of iPSC-derived MSCs

The iPSC cell line utilized for derivation of mesenchymal stem cells (iMSCs) was generated from neonatal human dermal fibroblasts as described previously [13]. Briefly, to isolate the iMSCs, iPSCs were differentiated *via* embryoid bodies (EBs) and attached onto culture dish to enable outgrowth of cells. The cells were isolated and expanded in DMEM (Sigma-Aldrich, St. Louise, MO, USA) supplemented with 10% foetal bovine serum (Hyclone. Thermo Fisher Scientific Inc, Waltham, MA, USA) he expanded cells were shown to express ∼90% positive surface markers of CD29, CD44, CD73, CD90 and CD105, which are characteristic markers for MSC. The derived iMSCs expanded exponentially up to 20 passages, retained stable telomerase activity and demonstrated multipotent capacity *via* directed differentiation into adipocytes/oesteocytes/chondrocytes as described [13]. Early passage iMSCs (up to passage 10) were used for subsequent experiments.

### Animal surgery and transplantation

The animal study was approved by the Singapore General Hospital IACUC committee and conformed with the Guide for the Care and Use of Laboratory Animals published by the US National Institutes of Health (NIH Publication No.85-23, revised 1996). Healthy female mice with severe combined immune deficiency (SCID) (10–12 weeks of age, weight 20–25 g) were used. Mice were induced and intubated with 1.5–2% isoflurane while connected to a small animal ventilator (model 687; Harvard Apparatus, Holliston, MA, USA) with a stroke volume (SV) of 0.3–0.5 ml/min. and respiration rate of 120 beats/min. All surgical manipulations were performed under a Leica stereotactic operation microscope on a heated surgical pad (Harvard Apparatus) at 37°C. A left thoracotomy was performed in the fourth intercostal space to assess anterior wall of the heart and the left anterior coronary artery was identified distal to the level of left atrium followed by passing a 7-0 suture (Ethicon, Johnson and Johnson, New Brunswick, NJ, USA) underneath the artery. The animals (*n* = 24) were randomly divided into three groups: sham-operated animals with mock infarction without ligating the suture over left coronary artery, saline-injected animals that received serum-free DMEM media (50 μl) or iMSC-injected animals that received 2 × 10^5^ cells in serum-free media (50 μl) intramyocardially into the LV free wall bordering the infarct zone *via* a 29G needle at approximately 45° canted angle, after the left coronary artery was permanently ligated. Immediately following ligation, occlusion was confirmed by observation of the LV pallor. The animal was gradually weaned off the ventilator until fully recovered and had free access to standard chow and water until next follow-up.

### Echocardiography and 2D speckle tracking analysis

Echocardiography was performed at baseline, 1-week and 8-week after infarction on Vevo2100 (VisualSonics VSI, Toronto, ON, Canada) with MS400 linear array transducer (38 MHz) by using optimized sector width for complete myocardial visualization (50 and 110 μm axial and lateral resolution respectively) and endocardial definition. A 2D guided M-mode of parasternal short axis at papillary muscle level was obtained to measure standard parameters. Long-axis view was used to obtain fractional area change (FAC = Simpson area_Ed_ − Simpson area_ES_/Simpson area_Ed_), ventricular chamber volumes and left ventricular ejection fraction (LVEF) were derived by using a modified Quinones method [22]. Average of 10 cardiac cycles at each plane was stored in cineloop for subsequent offline analysis. Standard parasternal long axis and short axis at mid papillary muscle level views with frame rate more than 200 frames/sec. recommended for optimal speckle tracking analysis was used. Global peak radial (RS) and circumferential strain (CS) sampled from anterior, lateral, posterior, inferior, postero-septal and antero-septal segments were measured from the short-axis view. Global peak longitudinal strain (LS) was measured from anterior basal, mid, apical and posterior basal, mid, apical segments from long-axis view. Strain data were analysed by VevoStrain version 1.3.0 (VisualSonics VSI).

### Histology and immunostaining

The harvested hearts were cryo-processed by using OCT Tissue-Tek medium. Hearts were sectioned transversely from the basal part to the apex of the left ventricle by using a cryostat with 5-um thickness (Leica AG, Solms, Germany). Masson's Trichrome staining (Sigma-Aldrich) was performed to quantify infarct size. The percentage infarct scar size was estimated from infarct area over total LV area by using a calibrated M205 steromicroscope (Leica AG). Overnight incubation with antibody against human-specific Ku80 (Clone EPR3468; Abcam, Cambridge, MA, USA) was used to identify the transplanted human iMSC and antibody against CD34 (clone 581; BD Biosciences, San Jose, CA, USA) was used to identify myocardial interstitial cells in the mouse heart and antibodies against α-actinin and α-smooth muscle actin (SMA) were used to identify cardiac muscle and vascular smooth muscle cells respectively (Sigma-Aldrich). Signals visualization was performed with 3,3′-diaminobenzidine (DAB) before counterstaining nuclei with haematoxylin and brightfield images analysed on a micrometre calibrated M200 microscope (Carl Zeiss, Gottingen, Germany). For vascular angiogenesis, microvessels of less than 200-μm caliber in the peri-infarcted myocardium, away from the pericardium, were identified by using antibody against von Willebrand factor (Dako, Glostrup, Denmark) and mature vessels were confirmed by co-staining by using antibody against α-SMA (Sigma-Aldrich) and Alexa Fluor488/555-conjugated secondary antibodies (Life Technologies, Carlsbad, CA, USA) before counterstaining the nuclei with 4′,6-diamidino-2-phenylindole (DAPI) and signals visualized by using M200 fluorescent microscope (Carl Zeiss). For co-staining of Ku80 identified human cells in histology sections (visualized by using DAB colorimetric method), sequential staining with primary antibody against CD34, α-actinin or α-SMA was followed by fluorescent secondary antibody.

### Statistical analysis

Data were presented as mean ± SD. Global peak strain was calculated as the average of all measurable segments. anova was performed comparing among experimental groups followed by post hoc analysis by using Dunnett *t*-test for all comparisons while Tukey test was performed for segment by segment comparison of speckle strain and neovascularization analysis for normally distributed data. Analysis was performed with SPSS software (version 13, SPSS Inc, Chicago, IL, USA) with *P* < 0.05 considered statistically significant.

## Results

### Cardiac performance

The proliferative and multipotent property of the iMSCs have been previously characterized by our group where successive expansion rapidly yielded large quantities of iMSCs in culture while adult BM-MSCs lost such potency with rapid telomere shortening [13]. Those expanded iMSC retained spindle morphology and MSC characteristics throughout passages before myocardial transplantation into peri-infarct region of acutely infarcted SCID mice.

**Fig. 1:**
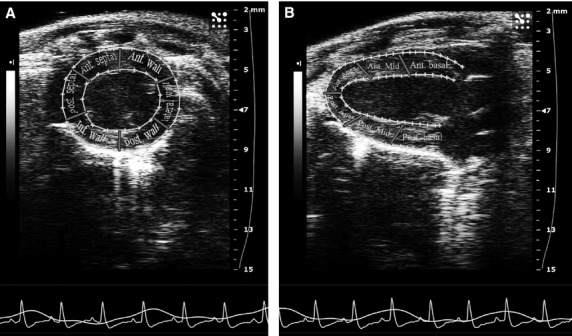
Short-axis and long-axis views of speckle tracking echocardiography. (**A**) Six-segment view from short axis of a mouse heart. (**B**) Six-segment view from long axis of a mouse heart. Ant.: anterior; post.: posterior; Inf.: inferior.

**Fig. 2:**
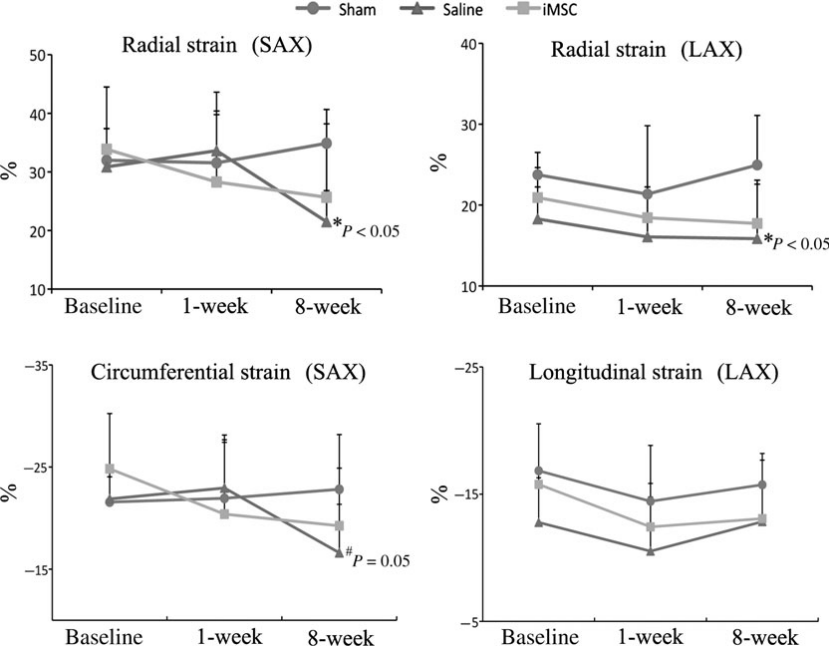
Global peak strain analysis of LV systolic deformations at baseline and follow-up. SAX: short-axis view; LAX: long-axis view.

There were no significant differences in all cardiac indices examined at 1-week afetr infarction among the sham-operated, saline-injected or iMSC-injected groups (Table 1). However, when compared to sham-operated control (3.74 ± 0.32 mm), transplantation of iMSC (4.24 ± 0.58 mm) into the peri-infarct region salvaged ventricular chamber geometry while saline-injection (4.40 ± 0.62 mm, *P* < 0.05) failed to restrict myocardial dilatation at 8-week after intervention. Furthermore, speckle tracking analysis of the regional wall in parasternal short-axis view (Fig. 1A) showed that iMSC (25.67 ± 12.53%) protected mean radial strain (six segments of anterior, lateral, posterior, inferior, posteroseptal and anteroseptal walls) of the left ventricle while functional deterioration was observed in saline-injected (21.50 ± 10.07%, *P* < 0.05) animals (Fig. 2, Table S1). This protected contractility was further corroborated by findings from the parasternal long-axis view (Fig. 1B) whereby iMSC transplanted animals (17.72 ± 4.86%) preserved ventricular radial strain, but not saline-injection (15.84 ± 7.24%, *P* < 0.05), when compared to sham-operated (24.95 ± 6.13%) animals (Fig. 2, Table S1). Nevertheless, neither iMSC (33.19 ± 10.99%, *P* < 0.05) nor saline-injection (32.96 ± 14.23%, *P* < 0.05) rescued the compromised LVEF and FAC when compared to sham-operated (51.17 ± 11.69%) animals (Table 1).

**Table 1:** Cardiac hemodynamics assessment by 2-D echocardiography

	Baseline			1-week			8-week		
			
2D Echo	Sham (*n* = 8)	Saline (*n* = 8)	iMSC (*n* = 8)	Sham (*n* = 8)	Saline (*n* = 8)	iMSC (*n* = 8)	Sham (*n* = 8)	Saline (*n* = 8)	iMSC (*n* = 8)
LVWTed (mm)	0.73 ± 0.07	0.77 ± 0.11	0.72 ± 0.06	0.79 ± 0.18	0.89 ± 0.14	0.84 ± 0.12	0.84 ± 0.17	0.85 ± 0.19	0.76 ± 0.15
LVIDed (mm)	3.54 ± 0.14	3.62 ± 0.23	3.35 ± 0.22	3.65 ± 0.36	3.95 ± 0.41	3.65 ± 0.36	3.74 ± 0.32	4.40 ± 0.62*	4.24 ± 0.58
LVWTes (mm)	1.05 ± 0.11	1.06 ± 0.16	1.04 ± 0.17	1.08 ± 0.20	1.22 ± 0.26	1.08 ± 0.20	1.12 ± 0.23	1.09 ± 0.31	1.05 ± 0.30
LVIDes (mm)	2.36 ± 0.21	2.61 ± 0.33	2.18 ± 0.40	2.56 ± 0.50	2.67 ± 0.47	2.73 ± 0.46	2.62 ± 0.58	3.40 ± 0.71	3.25 ± 0.81
EDV (μl)	45.43 ± 4.74	47.93 ± 7.05	45.22 ± 4.97	53.57 ± 11.00	64.40 ± 19.23	58.15 ± 19.26	57.28 ± 8.72	81.33 ± 31.34	76.24 ± 29.03
ESV (μl)	21.59 ± 2.91	25.66 ± 6.77	21.19 ± 2.53	29.59 ± 11.75	43.12 ± 19.28	40.27 ± 18.99	27.97 ± 9.75	57.68 ± 30.22	53.41 ± 29.14
FS (%)	35.33 ± 3.67	27.94 ± 5.66	35.34 ± 8.42	30.37 ± 8.95	32.67 ± 6.70	25.41 ± 8.27	30.62 ± 9.55	23.31 ± 6.44	24.36 ± 9.41
LVEF (%)	52.41 ± 5.33	47.38 ± 10.46	52.89 ± 5.94	45.91 ± 12.67	35.78 ± 13.28	33.46 ± 11.65	51.17 ± 11.69	32.96 ± 14.23*	33.19 ± 10.99*
FAC (%)	35.33 ± 3.67	32.13 ± 7.69	35.00 ± 4.83	31.38 ± 9.33	24.13 ± 8.63	21.63 ± 7.93	34.86 ± 9.82	21.50 ± 10.07*	21.25 ± 7.55*

* *P* < 0.05 *versus* sham-operated control.

LVWTed/es: left ventricle wall thickening at end diastole/systole; LVIDed/es: left ventricle internal dimension at end diastole/systole; EDV: end diastolic volume; ESV: end systolic volume; FS: fractional shortening; LVEF: left ventricular ejection fraction; FAC: fractional area change.

Segmental short-axis speckle tracking analysis revealed that ventricular radial strain at the lateral, posterior, inferior and posteroseptal walls were significantly compromised in the saline-injected animals while only posterior wall strain remained depressed in the iMSC-injected animals at 8-week afetr MI (Table 2). The ligation infarcted anterior wall that received iMSC showed better radial strain, although there was no significant difference between the iMSC and saline-injected animals. Nevertheless, segmental long-axis speckle tracking analysis of radial strain revealed a significant improvement in the anterior basal segment that coincided with iMSC-injected territory (Table 3). Circumferential and longitudinal strain were minimally affected whereby only inferior (circumferential strain) wall (−17.22 ± 7.26% *versus* −24.41 ± 5.62%, *P* < 0.05) of saline-injected (Table 2) and posterior apical (longitudinal strain) wall (−10.58 4.96% *versus* −23.63 8.55%, *P* < 0.05) of iMSC-injected animals (Table 3) remained significantly difference than sham-operated control at 8-week after infarction.

**Table 2:** Segmental analysis of myocardial strain in short axis

	8-week			
	
Speckle tracking analysis	Sham (*n* = 8)	Saline (*n* = 8)	iMSC (*n* = 8)	Stats sig.
*Short-axis view (SAX)*				
Mean radial strain (RS; %)	34.88 ± 5.77	21.50 ± 5.31*	25.67 ± 12.53	*P* < 0.05
Anterior wall	27.46 ± 8.71	20.53 ± 10.65	26.60 ± 18.99	NS
Lateral wall	35.96 ± 8.35	19.33 ± 8.73*	23.53 ± 23.51	*P* < 0.05
Posterior wall	41.49 ± 8.91	25.34 ± 9.84*	23.26 ± 15.12*	*P* < 0.05
Inferior wall	42.98 ± 11.59	25.19 ± 14.39*	28.62 ± 9.87	*P* < 0.05
Posterior septal wall	32.12 ± 6.61	20.44 ± 5.89*	25.76 ± 10.09	*P* < 0.05
Anterior septal wall	29.69 ± 9.04	18.35 ± 6.08	25.31 ± 11.64	NS
Mean circumferential strain (CS; %)	−22.82 ± 5.36	−16.61 ± 4.74^#^	−19.25 ± 5.64	*P* = 0.051
Anterior wall	−16.23 ± 5.33	−13.21 ± 5.18	−15.65 ± 7.01	NS
Lateral wall	−21.82 ± 9.15	−16.73 ± 4.54	−15.63 ± 8.86	NS
Posterior wall	−21.32 ± 4.62	−16.33 ± 6.54	−16.53 ± 7.24	NS
Inferior wall	−24.41 ± 5.62	−17.22 ± 7.26*	−22.12 ± 7.72	*P* < 0.05
Posterior septal wall	−32.01 ± 7.50	−21.12 ± 8.29	−25.75 ± 5.55	NS
Anterior septal wall	−19.94 ± 8.33	−15.53 ± 10.33	−18.18 ± 4.70	NS

**Table 3:** Segmental analysis of myocardial strain in long-axis view

	8-week			
	
Speckle tracking analysis	Sham (*n* = 8)	Saline (*n* = 8)	iMSC (*n* = 8)	Stats sig.
*Long-axis view (LAX)*				
Mean radial strain (RS; %)	24.95 ± 6.13	15.84 ± 7.24*	17.72 ± 4.86	*P* < 0.05
Posterior basal wall	21.93 ± 3.84	21.26 ± 14.88	22.17 ± 12.37	NS
Posterior mid wall	24.18 ± 8.19	19.72 ± 12.87	12.85 ± 10.57	NS
Posterior apical wall	21.18 ± 7.98	11.74 ± 9.72	10.60 ± 5.38	NS
Anterior basal wall	22.18 ± 4.13	15.81 ± 9.83	28.86 ± 8.16*	*P* < 0.05
Anterior mid wall	27.35 ± 9.81	15.18 ± 13.92	20.14 ± 14.19	NS
Anterior apical wall	29.55 ± 11.61	12.92 ± 13.22	14.78 ± 15.45	NS
Mean longitudinal strain (LS; %)	−15.74 ± 2.46	−12.84 ± 4.83	−13.08 ± 2.55	NS
Posterior basal wall	−12.66 ± 5.11	−18.53 ± 4.19	−14.18 ± 6.72	NS
Posterior mid wall	−16.59 ± 3.32	−10.71 ± 5.99	−11.71 ± 6.74	NS
Posterior apical wall	−23.63 ± 8.55	−13.00 ± 9.58	−10.58 ± 4.96*	*P* < 0.05
Anterior basal wall	−14.41 ± 5.14	−18.02 ± 5.18	−20.17 ± 6.03	NS
Anterior mid wall	−10.05 ± 3.87	−7.64 ± 4.32	−12.15 ± 4.62	NS
Anterior apical wall	−15.66 ± 6.48	−11.44 ± 6.59	−10.16 ± 5.20	NS

### Localization of transplanted iMSC

**Fig. 3:**
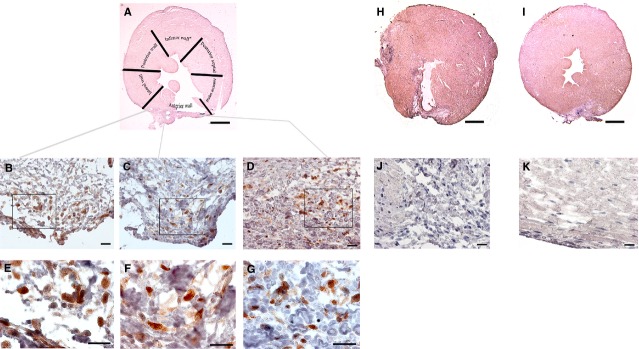
Myocardial localization of transplanted human iMSC in the anterior segment of left ventricle. (**A**) Six-segment view of transverse sectioned mouse heart injected with iMSCs. (**B**) Human-specific nuclear staining of Ku80 in iMSCs in peri-infarcted zone of anterior segment bordering lateral wall. (**C**) Human-specific nuclear staining of Ku80 in iMSCs in peri-infarcted zone of anterior segment next to infarcted wall. (**D**) Identification of human Ku80 stained iMSCs in the infarcted zone of anterior wall. (**E**–**G**) Magnified views of boxed region in **B**, **C** and **D**. (**H**) Transverse sectioned view of saline-injected mouse heart. (**J**) Magnified view of infarct border in **H**. (**I**) Transverse sectioned view of sham-operated mouse heart. (**K**) Magnified view of injured border in **I**. Scale bar: 1 mm (**A**, **H** and **I**); 20 μm (for all others).

**Fig. 4:**
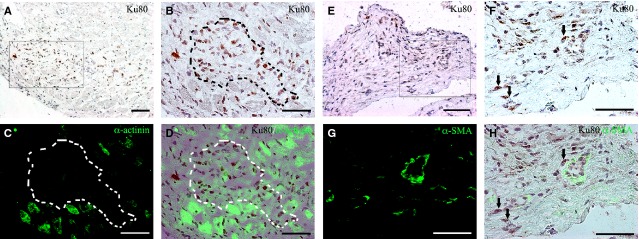
Interstitial localization of transplanted human iMSCs in myocardium. (**A**) Presence of human-specific nuclear staining of Ku80 iMSCs in the interstitial space of cardiac muscle. (**B**–**D**) Magnified view of boxed region in **A**, showing Ku80 stained iMSCs located mainly in the interstitial space (demarcated line) of α-actinin (green) stained cardiac muscle. (**E**) Presence of human-specific nuclear staining of Ku80 iMSCs in fibrous/collagenous region of myocardium. (**F**–**H**) Magnified view of boxed region in **E**, showing Ku80 stained human iMSCs (arrows) located in peri-vascular space of α-smooth muscle actin (SMA) stained microvessels; scale bar: 50 μm.

In examining the cross section tissue histology of the injected heart (Fig. 3A), transplanted iMSC were found mostly localized within the injected anterior wall in the peri-infarcted zone (Fig. 3B and C) bordering the lateral wall as well as in the infarcted zone (Fig. 3D). The iMSC that stained positive for human-specific Ku80 nuclear marker (Fig. 3E–G) were scattered among host cardiac fibroblasts and myocardial interstitial cells within the actively remodelling regions of anterior epicardium and myocardium. Rare iMSC were occasionally found in the endocardium of anterior wall region and sparingly as isolated single cells in the lateral wall that borders the anterior wall. Expectedly, no Ku80 positive human cells were detected in the saline-injected or sham-operated heart (Fig. 3H and I). Consistent with their interstitial localization, the Ku80 positive transplanted iMSCs (Fig. 4A, B and E) did not show appreciable differentiation towards cardiomyocytes (Fig. 4C and D) or vascular smooth muscle or endothelial cells (Fig. 4G and H).

**Fig. 5:**
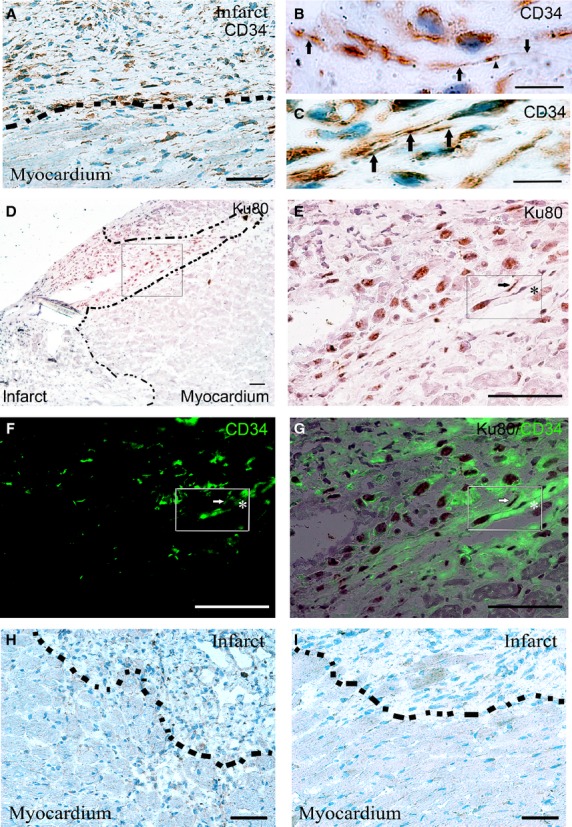
Presence of CD34 stained interstitial cells in the infarct zone. (**A**) Interstitial cells with long cellular processes that stained positive for CD34 that resided longitudinally in the collagen-rich and remodelling infarct zone of iMSC transplanted heart. (**B** and **C**) Magnified view of CD34 stained cells with thin and long cellular processes resembled podomers (arrows) and podom (arrowhead) of myocardial telocytes in the interstitial space. (**D**) Presence of human-specific nuclear staining of Ku80 iMSCs and interstitial cells in peri-infarct zone. (**E**–**G**) Magnified view of boxed region in **D**, showing Ku80 and CD34 double-stained human telocyte (arrow) in close proximity with CD34 stained (but human Ku80 negative) resident telocyte (*). (**H**) Absence of CD34 stained interstitial cells in the infarct zone of saline-injected mouse heart. (**I**) Absence of CD34 stained interstitial cells in the injured zone of sham-operated mouse heart. Dotted line demarcates intact myocardium from infarct zone. Scale bar: 50 μm (**A**, **D**–**I**); 10 μm (**B** and **C**).

Coincided with the presence of human Ku80 positively identified iMSC in the injected anterior wall, CD34 stained interstitial cells (Fig. 5A) with long cellular processes that resembled telopodes (Fig. 5B) of reported myocardial telocytes [6,23] were found nestled intimately with other interstitial cells mostly in the collagen-rich extracellular environment within the infarcted zone (Fig. 5C). Furthermore, such CD34^+^ telocytes were positive for human-specific Ku80 antigen (Fig. 5D and E) and were found to intermingle with resident CD34^+^ myocardial telocytes that were negative for the human antigen (Fig. 5F and G). In comparison, such distinct staining for CD34^+^ telocytes was mostly absent in the interstitial environment of infarcted anterior wall of saline-injected or sham-operated animals (Fig. 5H and I).

### Infarct size and vascular density

**Fig. 6:**
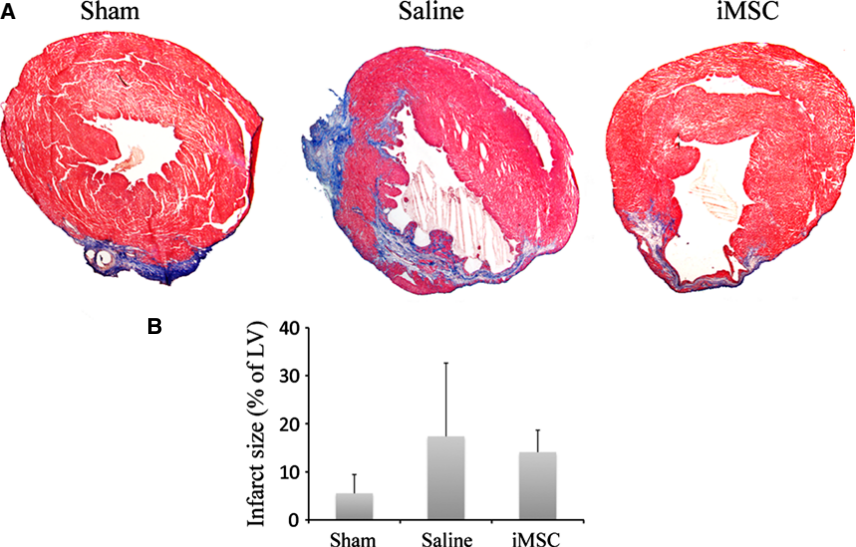
Infarct staining with Masson's trichrome staining. (**A**). Sham-operated mouse heart showing epicardial injury with limited fibrotic response and transmural anterior infarct with wall thinning and myocardial fibrosis in saline-injected and iMSC-injected mouse heart. (**B**) Quantitative estimation of infarct size at 8-week after infarction; scale bar: 1 mm.

In comparison to sham-operated animals (5.53 ± 3.91%) that showed mostly epicardial fibrotic response as a result of mock ligation-induced injury and no visible thinning of the anterior wall of the left ventricle (Fig. 6A), both iMSC-injected and saline-injected animals sustained larger transmural infarct and anterior wall thinning. Though smaller in severity, infarct size in animals that received iMSCs (14.08 ± 4.59%) was statistically insignificant when compared to saline-injected (17.36 ± 15.26%) animals (Fig. 6B).

**Fig. 7:**
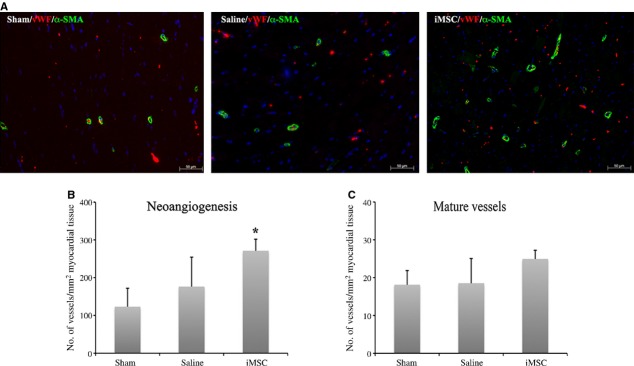
Microvascular neoangiogenesis at 8-week after myocardial infarction. (**A**) Immunofluorescent staining for von Willebrand (vWF) and a-smooth muscle actin (SMA) in left ventricle post-infarction. (**B**) Vascular density counts of vWF stained microvasculature in the left ventricle. (**C**) Vascular maturity estimated from vWF/SMA co-stained microvessels in the left ventricle; scale bar: 50 μm.

Consistent with better regional contractility following iMSC transplantation, there was a significantly higher vascular density count in the iMSC-injected (271.25 ± 30.38 microvessels/mm^2^ myocardial tissue, *P* < 0.05) animals as compared to the saline-injected (176.29 ± 77.99 microvessels/mm^2^ myocardial tissue) and sham-operated (123.02 ± 48.84 microvessels/mm^2^ myocardial tissue) animals at 8-week after infarction (Fig. 7A and B). Furthermore, the newly formed microvessels in the iMSC-injected (24.92 ± 2.32 α-SMA^+^ vessels/mm^2^ myocardial tissue, *P* < 0.05), but not the saline-injected (18.53 ± 6.54 α-SMA^+^ vessels/mm^2^ myocardial tissue) animals, were significantly more matured microvasculature accompanied by α-SMA as compared to the sham-operated (18.12 ± 3.75 α-SMA^+^ vessels/mm^2^ myocardial tissue) animals (Fig. 7C).

## Discussion

Myocardial transplantation of human BM-MSCs has been widely reported to promote functional recovery following MI. Consistently, improved cardiac function was observed in our study, despite only a limited number of injected iPSC-MSCs remained in the transplanted mouse heart by 8-week after AMI. Similar observations were reported in myocardial transplantation of adult BM-MSCs [24]. Although it was reported that ESC-derived MSCs were more cardiomyogenic and angiogenic than BM-MSCs [25], there was no appreciable difference in infarct size and no distinct evidence to support cardiac or endothelial differentiation of the transplanted iMSCs in our study.

Consistent with depressed radial strain in remote regions observed after anterior infarction [26], segmental short-axis analysis of the speckle tracking showed that ventricular radial strain at the lateral, inferior and posteroseptal walls were significantly compromised in the saline-injected, but not iMSC-injected, animals at 8-week after MI. Furthermore, strain analysed from the long-axis view, which was considered to be a better systolic contractility surrogate than LVEF [27,28], consistently showed enhanced strain in anterior wall that received iMSCs. This is consistent with observations in the POSEIDON trial, which showed regional contractility improvement in remote segments aside from MSC-injected segments in patients with advanced ventricular dysfunction [29].

The increased microvascular counts and enhanced vessel maturation in peri-infarct zones of iMSC-injected group suggested that paracrine-driven effect could have contributed partly to the positive outcome despite no appreciable iMSCs were found in the neighbouring ventricular segments. Nevertheless, the multi-segmental recovery in radial strain following iMSC injection into the anterior segment suggested that induced neoangiogenesis alone may not be responsible for the observed recovery in remote segments of the myocardium. Similar to previous reports by Berry *et al*. [17] and our group [30], reduction in wall stress and moderation of tissue stiffness in the infarcted anterior wall following cell transplantation may have resulted in overall geometry stabilization and functional preservation of other segments of the ventricular wall. This was further supported by the presence of telocytes in the interstitial space of infarcted anterior wall segment in iMSC-injected heart, which was consistent with their reported roles in co-ordinating and supporting architectural organization, tissue elasticity and mechanotransduction across network of myocardial segments [9,10,31]. Indeed, preservation of ventricular wall mechanics and structural organization for cyclical laminar shearing and extension experienced throughout systole and diastole are known to be critical in regional mechanical function of the heart [32]. Interestingly, the declining myocardial strain observed between 1-week and 8-week follow-up in the saline-injected group coincided with the period of reduced presence of cardiac telocytes following MI reported previously in left anterior descending (LAD) artery ligated rodent [33]. Consistently, presence of putative telocytes in the infarcted anterior segment that received iMSC transplantation, but not saline-injection, may be important in cardiac function improvement observed in our study. Consistent with beneficial effect of telocyte transplantation in recovering cardiac function [18], this was likely through sustaining the lattice of mechanical and biological interconnectivity from infarcted segment to other remote segments of the myocardium as demonstrated by close association of exogenous telocytes with resident telocytes observed in the infarcted and peri-infarcted zones.

Analysis of the speckle strain from short-and long-axis views revealed that peri-infarct injection of iMSCs into the anterior wall benefited mostly radial strain (*P* < 0.05), but their effect was marginal in circumferential (*P* = 0.051) and limited in longitudinal (*P* = NS) strain. However, influence of iMSC injection angle (at 45° angle to the heart wall) in connection with outcome observed in radial strain cannot be totally discounted. Furthermore, it remains to be ascertained if torsional mechanics of the ventricular wall, which is known to intertwine with other segmental strain in affecting systolic deformation [34], had any role in the observed strain improvements in neighbouring and remote ventricular segments following iMSC injection.

In summary, our results show that iPSC-derived hMSCs ameliorate MI-associated ventricular remodelling and preserve global myocardial strain *via* paracrine-driven effect by enhancing neoangiogenesis and promoting myocardial deformation/compliance *via* parenchymal and interstitial cell interactions in the infarcted myocardium.

## Supporting information

**Table S1** Speckle tracking echocardiography of myocardial strain showing iMSC preserved myocardial function.

## References

[1] Miettinen JA, Ylitalo K, Hedberg P (2010). Determinants of functional recovery after myocardial infarction of patients treated with bone marrow-derived stem cells after thrombolytic therapy. Heart.

[2] Hare JM, Traverse JH, Henry TD (2009). A randomized, double-blind, placebo-controlled, dose-escalation study of intravenous adult human mesenchymal stem cells (prochymal) after acute myocardial infarction. J Am Coll Cardiol.

[3] Chen SL, Fang WW, Qian J (2004). Improvement of cardiac function after transplantation of autologous bone marrow mesenchymal stem cells in patients with acute myocardial infarction. Chin Med J (Engl).

[4] Mehta A, Shim W (2013). Cardiac stem cell therapy: stemness or commitment?. Cell Transplant.

[5] Zheng Y, Cretoiu D, Yan G (2014). Comparative proteomic analysis of human lung telocytes with fibroblasts. J Cell Mol Med.

[6] Diaz-Flores L, Gutierrez R, Garcia MP (2014). CD34+ stromal cells/fibroblasts/fibrocytes/telocytes as a tissue reserve and a principal source of mesenchymal cells. Location, morphology, function and role in pathology. Histol Histopathol.

[7] Popescu LM, Gherghiceanu M, Manole CG (2009). Cardiac renewing: interstitial Cajal-like cells nurse cardiomyocyte progenitors in epicardial stem cell niches. J Cell Mol Med.

[8] Manole CG, Cismasiu V, Gherghiceanu M (2011). Experimental acute myocardial infarction: telocytes involvement in neo-angiogenesis. J Cell Mol Med.

[9] Popescu LM, Manole CG, Gherghiceanu M (2010). Telocytes in human epicardium. J Cell Mol Med.

[10] Bani D, Formigli L, Gherghiceanu M (2010). Telocytes as supporting cells for myocardial tissue organization in developing and adult heart. J Cell Mol Med.

[11] Gherghiceanu M, Manole CG, Popescu LM (2010). Telocytes in endocardium: electron microscope evidence. J Cell Mol Med.

[12] Lai RC, Choo A, Lim SK (2011). Derivation and characterization of human ESC-derived mesenchymal stem cells. Methods Mol Biol.

[13] Wei H, Tan G, Manasi (2012). One-step derivation of cardiomyocytes and mesenchymal stem cells from human pluripotent stem cells. Stem Cell Res.

[14] Simpson DL, Boyd NL, Kaushal S (2012). Use of human embryonic stem cell derived-mesenchymal cells for cardiac repair. Biotechnol Bioeng.

[15] Lai RC, Arslan F, Lee MM (2010). Exosome secreted by MSC reduces myocardial ischemia/reperfusion injury. Stem Cell Res.

[16] Hansson EM, Lindsay ME, Chien KR (2009). Regeneration next: toward heart stem cell therapeutics. Cell Stem Cell.

[17] Berry MF, Engler AJ, Woo YJ (2006). Mesenchymal stem cell injection after myocardial infarction improves myocardial compliance. Am J Physiol Heart Circ Physiol.

[18] Zhao B, Liao Z, Chen S (2014). Intramyocardial transplantation of cardiac telocytes decreases myocardial infarction and improves post-infarcted cardiac function in rats. J Cell Mol Med.

[19] Bauer M, Cheng S, Jain M (2011). Echocardiographic speckle-tracking based strain imaging for rapid cardiovascular phenotyping in mice. Circ Res.

[20] Teske AJ, De Boeck BW, Melman PG (2007). Echocardiographic quantification of myocardial function using tissue deformation imaging, a guide to image acquisition and analysis using tissue Doppler and speckle tracking. Cardiovasc Ultrasound.

[21] Mondillo S, Galderisi M, Mele D (2011). Speckle-tracking echocardiography: a new technique for assessing myocardial function. J Ultrasound Med.

[22] Quinones MA, Waggoner AD, Reduto LA (1981). A new, simplified and accurate method for determining ejection fraction with two-dimensional echocardiography. Circulation.

[23] Popescu LM, Gherghiceanu M, Hinescu ME (2006). Insights into the interstitium of ventricular myocardium: interstitial Cajal-like cells (ICLC). J Cell Mol Med.

[24] Simpson D, Liu H, Fan TH (2007). A tissue engineering approach to progenitor cell delivery results in significant cell engraftment and improved myocardial remodeling. Stem Cells.

[25] Ramkisoensing AA, Pijnappels DA, Askar SF (2011). Human embryonic and fetal mesenchymal stem cells differentiate toward three different cardiac lineages in contrast to their adult counterparts. PLoS ONE.

[26] Migrino RQ, Zhu X, Morker M (2008). Myocardial dysfunction in the periinfarct and remote regions following anterior infarction in rats quantified by 2D radial strain echocardiography: an observational cohort study. Cardiovasc Ultrasound.

[27] Reisner SA, Lysyansky P, Agmon Y (2004). Global longitudinal strain: a novel index of left ventricular systolic function. J Am Soc Echocardiogr.

[28] Antoni ML, Mollema SA, Delgado V (2010). Prognostic importance of strain and strain rate after acute myocardial infarction. Eur Heart J.

[29] Suncion VY, Ghersin E, Fishman J (2014). Does transendocardial injection of mesenchymal stem cells improve myocardial function locally or globally? An analysis from the POSEIDON randomized trial. Circ Res.

[30] Qian L, Shim W, Gu Y (2012). Hemodynamic contribution of stem cell scaffolding in acute injured myocardium. Tissue Eng Part A.

[31] Kostin S, Popescu LM (2009). A distinct type of cell in myocardium: interstitial Cajal-like cells (ICLCs). J Cell Mol Med.

[32] Coppola BA, Omens JH (2008). Role of tissue structure on ventricular wall mechanics. Mol Cell Biomech.

[33] Zhao B, Chen S, Liu J (2013). Cardiac telocytes were decreased during myocardial infarction and their therapeutic effects for ischaemic heart in rat. J Cell Mol Med.

[34] Smerup M, Partridge J, Agger P (2013). A mathematical model of the mechanical link between shortening of the cardiomyocytes and systolic deformation of the left ventricular myocardium. Technol Health Care.

