# Supplementary material for: iPSC-derived human mesenchymal stem cells improve myocardial strain of infarcted myocardium
Source: J Cell Mol Med. 2014 Jun 28;18(8):1644–54. doi: 10.1111/jcmm.12351 (PMC4190910; doi:10.1111/jcmm.12351)
Supplement: Supplementary file 1 — Table S1 Speckle tracking echocardiography of myocardial strain showing iMSC preserved myocardial function. [file jcmm0018-1644-SD1.doc]

|  |  | Baseline |  |  |  | 1-week |  |  |  | 8-week |  |
| --- | --- | --- | --- | --- | --- | --- | --- | --- | --- | --- | --- |
|  | Sham  n = 8 | Saline  n = 8 | iMSC  n = 8 |  | Sham  n = 8 | Saline  n = 8 | iMSC  n =8 |  | Sham  n = 8 | Saline  n = 8 | iMSC  n = 8 |
|  |  |  |  |  |  |  |  |  |  |  |  |
| Mean  RS SAX (%) | 31.99 ± 5.41 | 30.86/6.52 | 33.86/10.63 |  | 31.54/8.85 | 33.62/9.99 | 28.28/11.49 |  | 34.88/5.77 | 21.50/5.31* | 25.67/12.53 |
| Mean  CS SAX (%) | -21.57/2.48 | -21.88/3.14 | -24.83/5.41 |  | -21.94/6.20 | -22.95/4.72 | -20.39/7.03 |  | -22.82/5.36 | -16.61/4.74^#^ | -19.25/5.64 |
| Mean  RS LAX (%) | 23.75/2.76 | 18.28/3.96 | 20.92/3.72 |  | 21.35/8.46 | 16.06/5.10 | 18.43/3.81 |  | 24.95/6.13 | 15.84/7.24* | 17.72/4.86 |
| Mean  LS LAX (%) | -16.84/3.69 | -12.78/3.51 | -15.77/1.30 |  | -14.44/4.39 | -10.52/5.32 | -12.43/2.34 |  | -15.74/2.46 | -12.84/4.83 | -13.08/2.55 |
|  |  |  |  |  |  |  |  |  |  |  |  |

Supplementary Table 1. Speckle tracking echocardiography of myocardial strain showing iMSC preserved myocardial function. RS: radial strain, CS: circumferential strain, LS: longitudinal strain, SAX: short-axis, LAX: long-axis. *p<0.05 vs. Sham-operated control, ^#^p=0.05 vs. Sham-operated control.
